# Patient self-report section of the ASES questionnaire: a Spanish validation study using classical test theory and the Rasch model

**DOI:** 10.1186/s12955-016-0552-1

**Published:** 2016-10-18

**Authors:** Kalliopi Vrotsou, Ricardo Cuéllar, Félix Silió, Miguel Ángel Rodriguez, Daniel Garay, Gorka Busto, Ziortza Trancho, Antonio Escobar

**Affiliations:** 1 Unidad de Investigación de Atención Primaria-OSIS de Gipuzkoa, Instituto Biodonostia, Paseo Dr. Begiristain s/n, 20014 San Sebastián, Spain; 2 Red de Investigación en Servicios de Salud en Enfermedades Crónicas (REDISSEC), Bilbao, Spain; 3 Instituto de Investigación Sanitaria Biodonostia, San Sebastián, Spain; 4 Servicio de Traumatología y Cirugía Ortopédica, Hospital Universitario Donostia, San Sebastián, Spain; 5 Servicio de Traumatología y Cirugía Ortopédica, Hospital Universitario Basurto, Bilbao, Spain; 6 Servicio de Traumatología y Cirugía Ortopédica, Hospital Universitario Cruces, Barakaldo, Spain; 7 Servicio de Traumatología y Cirugía Ortopédica, Hospital Galdakao-Usansolo, Galdakao, Spain; 8 Servicio de Traumatología y Cirugía Ortopédica, Hospital de Mendaro, Mendaro, Spain; 9 Unidad de Investigación, Hospital Universitario Basurto, Bilbao, Spain

**Keywords:** ASES-p, Shoulder, Spanish validation, Constant Murley Score, SF-36, Validity, Responsiveness, Confirmatory factor analysis, Rasch model

## Abstract

**Background:**

The aim of the current study was to validate the self-report section of the American Shoulder and Elbow Surgeons questionnaire (ASES-p) into Spanish.

**Methods:**

Shoulder pathology patients were recruited and followed up to 6 months post treatment. The ASES-p, Constant, SF-36 and Barthel scales were filled-in pre and post treatment. Reliability was tested with Cronbach’s alpha, convergent validity with Spearman’s correlations coefficients. Confirmatory factor analysis (CFA) and the Rasch model were implemented for assessing structural validity and unidimensionality of the scale. Models with and without the pain item were considered. Responsiveness to change was explored via standardised effect sizes.

**Results:**

Results were acceptable for both tested models. Cronbach’s alpha was 0.91, total scale correlations with Constant and physical SF-36 dimensions were >0.50. Factor loadings for CFA were >0.40. The Rasch model confirmed unidimensionality of the scale, even though item 10 “do usual sport” was suggested as non-informative. Finally, patients with improved post treatment shoulder function and those receiving surgery had higher standardised effect sizes.

**Conclusions:**

The adapted Spanish ASES-p version is a valid and reliable tool for shoulder evaluation and its unidimensionality is supported by the data.

**Electronic supplementary material:**

The online version of this article (doi:10.1186/s12955-016-0552-1) contains supplementary material, which is available to authorized users.

## Background

Shoulder pathologies are among the commonest musculoskeletal problems, with subacromial pain, rotator cuff deficiencies, instability and fractures being some of the most frequent diagnoses [1–3]. Shoulder disorders are known to limit daily life activities [4], increase work absence [5] and affect psychological and social well-being [6]. A systematic review reported the estimated lifetime prevalence of shoulder pathologies in the general population between 69 and 667 per 1000 adults [7]. Different instruments exist for the assessment of the pathological shoulder, with the Constant-Murley score (CMS) being the most widely used scale for the functional assessment of this articulation [8]. The CMS is based on expert evaluation and measures pain level, activities of daily living (ADL), range of movement (ROM) and shoulder strength [9]. In addition, a big number of self-reported health related quality of life (HRQoL) shoulder scales can also be found in the literature. Some of them are pathology-specific, while others can be applied to any shoulder disorder. Among the most implemented such instruments is the American Shoulder and Elbow Surgeons patient self-report section (ASES-p) [8]. The ASES-p is an 11 item scale which evaluates pain level and 10 ADL activities. The full ASES questionnaire, originally published in 1994, additionally includes a specialist’s section, assessing ROM, strength, instability and other shoulder pathology signs, but a score index is only derived for the ASES-p section. As a result, the self-report part of the initial questionnaire has been used over the years independently of the specialist’s questions [10]. The properties of the ASES-p scale have been studied in different populations [11, 12] and the instrument has been culturally adapted and validated in several languages [13–18]. A standardized comparison of the psychometric properties of several shoulder HRQoL scales, indicated ASES-p as having the best overall rating [19].

Up to date, no Spanish language validation of this scale exists. The aim of the current study was to cross culturally adapt and validate the ASES-p questionnaire for its use in Spanish populations. To this end, an extensive validation was performed by applying both confirmatory factor analysis (CFA) and the Rasch model.

## Methods

### Cultural adaptation and pilot study

The cultural adaptation of the ASES-p questionnaire, from English to Spanish, was performed following the recommendations of the International Quality of Life Assessment (IQOLA) project. The IQOLA protocol is considered a reference standard for translating health status instruments [20, 21]. Two persons, an orthopaedic surgeon and a professional translator (not familiar with shoulder related pathologies), both native Spanish speakers independently translated the English version into Spanish. After discussing the conceptual equivalence of the two translations and resolving discrepancies, a consensus was reached for the first Spanish version of the ASES-p questionnaire. In a second phase, two professional translators, whose first language was English, back translated the first Spanish ASES-p version into English. Discrepancies were again discussed and resolved. The back translated English version was compared with the original ASES-p version, by the participating translators. Differences were discussed and corresponding changes were made in the Spanish version. One of the principal investigators (KV) participated in the discussions between the parts in all translation stages. A committee of two orthopaedic surgeons (DG, FS), one health professional (AE), a professional translator and KV accepted the pre-final translated version of ASES-p. In order to assess its comprehensiveness, this version was administered to a sample of *n* = 10 randomly chosen shoulder pathology patients. They were asked to fill-in the scale and comment on its understanding and item relevance. None of the pilot study patients were included in the validation study.

### Patient recruitment and data collection

Participants were recruited by the orthopaedic surgeons of five public hospitals, located in the Basque Country (Spain). Included patients were ≥18 years old, had a shoulder pathology, were going to receive a surgical or conservative treatment in the affected shoulder, and were able to speak and write in Spanish. Patients previously operated in the affected shoulder and those with cognitive impairment were excluded from the study.

Upon recruitment, functional assessment of the affected shoulder was performed by the orthopaedic surgeons, with the CMS instrument. Information on age, sex, marital status, daily lifestyle habits, medication consumption, additional pathologies and other questions of interest were filled in by the participants in their homes. The socio-demographic and clinical variables were sent by postal mail and replies were received in the same way. A reminder letter was sent to those not responding within two weeks, followed by further phone calls if necessary. If despite all efforts no reply was obtained, the patients were considered drop-outs. All assessments were performed twice: at recruitment and after the treatment. Conservatively treated and operated patients were assessed at 3 and 6 months respectively. Only one shoulder per patient was considered in the validation analyses.

### The ASES-p questionnaire

The ASES-p scale is composed of 11 items, divided in 2 subscales: pain (1 item) and function (10 items). The pain item evaluates current pain level on a 10 cm VAS with minimum and maximum values “0 = no pain at all” and “10 = pain as bad as it can be” respectively. The 10 function items evaluate the ability to perform certain daily life activities, and are answered on a 4 point Likert scale from “0 = unable to do” to “3 = not difficult”. Each subscale is assigned from 0 to 50 points, with higher values indicating better health status. Points are calculated as: (10-VAS × 5) and ((5/3) × sum of 10 function items) for the pain and function subscale respectively. The total ASES-p score is the sum of the two subscales, with possible values ranging between 0 and 100 points. The originally published form of the scale was implemented in this study [10].

For analyses where individual item replies were considered, the pain item was reversed. In order to ease interpretation, this item’s replies were transformed to be on the same direction with the function items (i.e. more points, better health). Maximum and no pain at all were thus given 0 and 10 points respectively. For certain analyses, the pain score was further categorized in four groups, approximating the 4 point Likert responses. In those cases, values 0–1 were considered as “3 = no pain”; values 2–5 were “2 = some pain”; 6–8 as “1 = a lot of pain” and values 9–10 as “0 = maximum pain”. This categorization was decided by examining this item’s responses in relation to pain and general health items of SF-36. All analyses are based on available data. No imputations have been performed in this study.

### Other measures

The Constant-Murley score (CMS) was published in 1987 and was approved by the executive committee of the European Society for Surgery of the Shoulder and the Elbow (ESSSE) [9, 22]. It assesses pain level, activities of daily living (ADL), range of movement (ROM) and strength, based on 2, 4, 4 and 1 item respectively. It is the most widely used questionnaire for the functional assessment of the shoulder and requires a specialist’s assessment [8]. In this study the ESSSE [23] CMS version was implemented. The strength component of CMS was assessed with the use of adjustable weights, while goniometers were used for the ROM items of flexion and abduction. The CMS questionnaire was filed in by the participating orthopaedic surgeons, all of whom were experienced in its administration. The Original CMS (CMS_O_) score assigns 15, 20, 40 and 25 points to each of its four components and its total score ranges between 0 and 100 points with higher scores indicating better shoulder function [9]. The relative CMS score adjusted for age and sex (CMS_R_) as suggested by the original CMS author [24] and an additional CMS, excluding the strength component (CMS_NS_) [25] were also implemented in this study.

The 36-Item Short Form health Survey (SF-36) [26] is a generic instrument composed of 36 items assessing eight dimensions: physical functioning, role physical, bodily pain, general health, vitality, social functioning, role emotional and mental health. The 8 dimensions are further grouped into two summary components: a physical (PCS) and a mental (MCS) one. Dimension and component summary scores range from 0–100 points. The SF-36 has been translated and validated into Spanish [27]. In this study the SF-36 v2 version was implemented and scores were derived using the Quality Metric Software (QualityMetric Health Outcomes™ Scoring Software 4.5.1). The Barthel Index [28, 29] was used for evaluating basic activities of daily living (BADL). The scale is composed of 10 items and its total score ranges from 0 to 100 points, indicating completely dependent and independent individuals respectively [30].

For surgically treated patients, data related to hospital admission and intervention of the affected side was gathered from the clinical history files. Patients not receiving any treatment during the study course were also assessed at the end of follow-up.

### Statistical analysis

Categorical data are presented with frequencies and percentages. Continuous data are presented with means and standard deviations (SD) when normally distributed, or medians and interquartile range (IQR) when skewed. Between-group comparisons of categorical and continuous data were performed with the chi-square, Student’s *t*-test or Wilcoxon rank-sum test respectively. Pre-post comparisons of the ASES-p scores performed with the paired *t*-test.

### Reliability

Reliability was estimated with Cronbach’s alpha [31]. Item-item and item-total correlations were estimated with Spearman’s correlation coefficient (r_S_). Values ≥0.70 and ≥30 respectively were considered acceptable [32]. Item-total correlations were controlled for overlapping, as total scores excluded the respective item (i.e. “rest” total score was implemented). Cronbach’s alpha was estimated considering both all ASES-p items (with pain as categorical) and the 10 function scale items.

### Validity

Construct validity was studied with two separate methods: confirmatory factor analysis (CFA) [33] and the Rasch model [34]. Even though the ASES-p is considered as having two components (pain and function), the fact that pain is evaluated with a single item does not allow neither for a two-factor CFA model, nor for Rasch unidimensionality to be tested per component. For this reason, in the present study 10 (function only) and 11 item (pain and function jointly) one latent factor models were fitted. CFA was performed with the unweigthed least squares (ULSMV) estimation method; recommended for ordinal or continuous non-normal indicators and samples <200 subjects [35]. In these models pain was implemented in its reversed form. Factor loadings ≥0.40 were considered acceptable [33]. The goodness of fit indexes examined were the root mean square error of approximation (RSMEA) with acceptable values <0.08; the Tucker-Lewis Index (TLI) and comparative fit index (CFI) with acceptable values >0.90 [33]. Residual values and modification indexes (MI) were examined. MI values ≥10 were considered for possible model modifications. The unidimensionality of the scale was additionally tested using the Rasch model, with pain as a categorical item. Difficulty estimations (logit) were derived and the infit and outfit mean square (MNSQ) statistics were explored. Desirable values for the latter lie between 0.6 and 1.4 [34]. Values <0.5 are less productive and those between 1.5 and 2.0 are unproductive for measurement construction, but none of them degrading; while values >2.0 are distorting the measurement system [36]. Person and item reliability indexes, as well as separation statistics were also examined with desired values being >0.80 and >2.0 respectively [34]. Point-measure correlations and average category measures were studied. Unidimensionality was further assessed via principal component analysis (PCA) of the Rasch model residuals. Lack of contrast eigenvalues ≥3 supports the unidimensionality of the scale. The Rasch-Andrich Rating Scale Model, for polytomous items was used [34, 37].

Convergent and divergent validity was explored via correlations with other scales. The ASES-p total score was correlated with the CMS, the SF-36 scale, and the Barthel index. It was hypothesized that ASES-p would present higher correlations (r_S_) with the CMS, the physical dimensions and physical component of SF-36, and Barthel. Lower correlations were expected with the mental dimensions and component of SF-36. Known-group validity was studied by examining the ASES-p score values against CMS, PCS and Barthel after transforming them to categorical variables. Comparisons were performed with the Jonckheere–Terpstra [38], testing for a trend among ordered categories and Student’s *t*-test when two categories where compared.

### Responsiveness to change

At follow up, patients were asked to evaluate whether their ROM and capacity in doing their ADL had improved, compared to baseline. Those with a positive reply to both questions were considered as improved. We hypothesized that these patients would present higher ASES-p pre-post score differences, compared to the rest. Three effect sizes were calculated: the standardized effect size (SES), the standardized response mean (SRM) and the SRM adjusted for paired observations (SRM_Adj_). The SES is the mean difference between baseline and follow-up scores divided by the SD of the baseline score [39]. The SRM is the mean difference between baseline and follow-up divided by the SD of the difference [39]; while the SRM_Adj_ was calculated considering the pooled effect size and the correlation of the respective pre-post observations [40]. Cohen’s definition about magnitude of effect sizes was considered, with values of 0.20, 0.50 and 0.80 perceived as small, medium and large [41]. Effect sizes were additionally studied considering three treatment groups: surgical intervention, infiltration and other.

### Sample size

Around 10 subjects per item are recommended for scale validations [33]. Based on previous experience with this kind of studies it was estimated that 30 % of the recruited subjects would not eventually participate. Given that the ASES-p consists of 11 items, a minimum of *N* = 160 subjects had to be recruited in the study.

Statistically significant results were considered those with *p*-values ≤ 0.05. Analyses were performed with the softwares of SAS (version 9.3; SAS Institute, Cary, NC), Mplus (version 7.4; Muthén et al., 1998-2015) and Winsteps (version 3.91.0.0; John M. Linacre, Chicago).

## Results

### Cultural adaptation and pilot study

During the translation-back-translation process of the ASES-p questionnaire, the only item that presented a certain difficulty was the fourth function item “manage toileting”. In the English-Spanish translation, one of the translators considered this item to refer to washing, dressing and attending one’s appearance, whereas the other perceived it as the difficulty one may have in cleaning oneself after urinating and defecating. By contacting the main author of one of the first ASES-p validations [11], it was clarified that the second definition was the correct one. No more important discrepancies existed and the back-translated version was found to be equivalent to the original. All pilot study subjects considered the adapted version easy to understand and none of the items were considered as non-relevant. Two of them left item 10 “do usual sport”, unanswered, for not being involved in any sport activities. No more changes were made in the Spanish ASES-p adapted version after the pilot study (Additional file 1).

### Baseline data

**Fig. 1:**
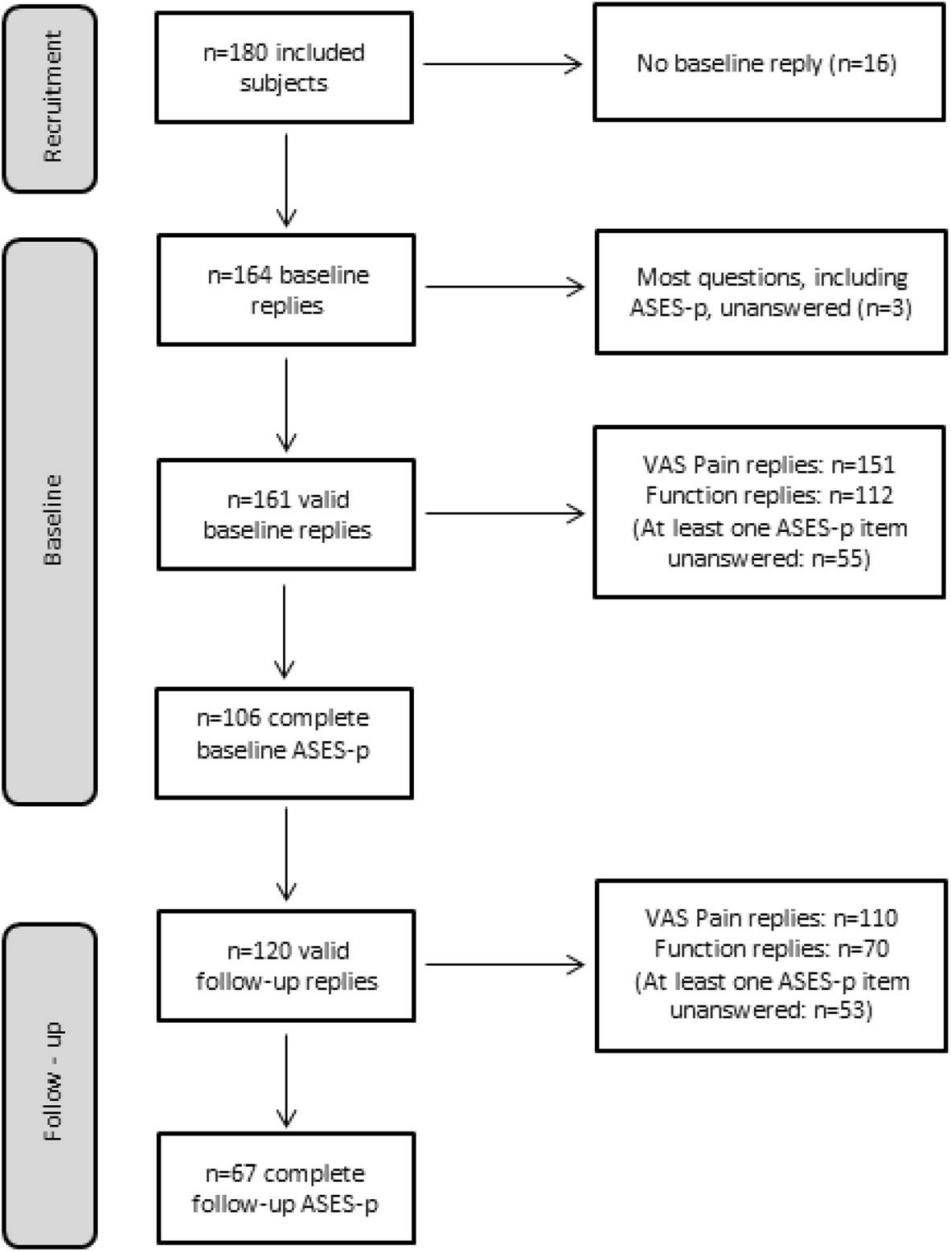
Flowchart of baseline and follow-up ASES-p received replies

Recruitment took place from May 2012 to November 2013 and the follow up was completed in June 2014. A total of *n* = 180 eligible subjects were recruited in the study and *n* = 164 returned the mailed baseline questionnaires. Three subjects who left all ASES-p items and many other questions unanswered, were excluded, leaving a total of *n* = 161 valid replies at baseline. Among these replies, missing data ranged from 1 to 6 % for most items, with the exception of item 10 “do usual sports”, which was not filled in by 23 % of the participants In total, *n* = 151 subjects replied to the pain item and *n* = 112 answered all function items. The ASES-p scale was fully answered by *n* = 106 subjects at baseline (Fig. 1).

Participants were between 22 and 82 years of age and half of them were females (51 %). The majority had subacromial pathology with rotator cuff rupture (64 %), presented mostly on the right shoulder (68 %). Thirty-five per cent of the responders were manual workers (heavy or light tasks); around one-third had taken at least one sick-leave due to their shoulder problem in the last 5 years and many suffered additionally by back (52 %), neck (47 %) or lower extremity (39 %) problems (Table 1).

**Table 1:** Baseline characteristics and ASES-p values

Variables	*N* = 161
Age in years; mean (SD)	59.7 (11.8)
Gender	
Female	82 (51)
Male	79 (49)
Affected shoulder	
Right	110 (68)
Left	51 (32)
BMI	
< 25	50 (32)
25–29.9	69 (44)
≥ 30	39 (25)
Years with problem; median (Q1, Q3)	3 (1, 7)
Smoking status	
Current smoker	31 (19)
Ex-smoker	48 (30)
Never smoked	82 (51)
Relationship status	
Married/living with couple	120 (75)
Single/divorced/separated	26 (16)
Widowed	14 (9)
Educational level	
Primary or less	67 (42)
Secondary	70 (44)
University or higher	22 (14)
Additional problems^a^	
Back	84 (52)
Neck	75 (47)
Lower extremity	63 (39)
Upper extremity	10 (6)
Type of work	
Manual	56 (35)
Office	26 (16)
Homemaker	31 (20)
Pensioner	36 (23)
Studying/unemployed	3 (2)
Sick-leave in past 5 years	
Yes	53 (33)
No	108 (67)
Diagnosis	
Subacromial path. with RC rupture	103 (64)
Subacromial path. without RC rupture	13 (8)
Tendinopathy: tendinitis/tendinosis	27 (17)
Instability: traumatic/non-traumatic	7 (4)
Arthrosis	5 (5)
Necrosis avascular	1 (1)
Frozen shoulder	2 (1)
ASES-p; mean (SD)^b^	
Total score (0–100 points)	46.5 (22.9)
Pain score (0–50 points)	23.8 (12.9)
Function score (0–50 points)	22.9 (11.9)

Numbers are: frequency (percentage), unless otherwise stated

*SD* standard deviation, *Q1, Q3* first and third quartile, *RC* rotator cuff

^a^A patient may have more than one additional pathologies, therefore sum is > N

^b^ASES-p scores are based on available data, total score: *n* = 106; pain: *n* = 151 and function: *n* = 112 subjects

The option “unable to do”, across all ASES-p items, was chosen by 5–56 % of the responders, while “not difficult” by 5–31 %. The mean scale value of the affected shoulder at baseline was 46.5 (SD: 22.9) points. Neither floor nor ceiling effects were observed for the total questionnaire score, as only 1 % of the participants obtained 5 and 100 points respectively. Eighty-two (51 %) of the responders provided valid ASES-p answers for the non-affected shoulders too. Based on these replies, the contralateral shoulder had a mean ASES-p of 66.3 (SD: 21.1) points, with the difference between the two sides being statistically significant (*p* < 0.0001). Sixty-six (*n* = 66) of the participants received a surgical intervention, while for *n* = 2 the intervention was still pending at the end of the study; *n* = 28 underwent infiltration; *n* = 8 rejected surgery and the rest underwent a rehabilitation program, followed by a health professional at hospital or at their home. The mean age of those not responding to item 10 was 64.9 (SD: 10.3) years. No differences in terms of age (*p* = 0.348), sex (*p* = 0.339) and the three CMS versions (*p* > 0.900) were found between responders and non-responders at baseline.

### Reliability

Cronbach’s alpha coefficients for the ASES-p scale were equal to 0.91, both when considering all scale items and after excluding the pain item. Item-item correlations were r_S_ >0.30, with only exception the correlations of item 10 “do usual sports” with pain level (r_S_ = 0.261) and item 3 “wash back” (r_S_ = 0.291). The item-scale total correlations, oscillated between 0.73 and 0.40, with the lowest value corresponding to item 10 (Table 2).

**Table 2:** Confirmatory factor analyses results and item-scale total correlations

Item no.	Description	All ASES-p items (*n* = 106)	Function items^b^ (*n* = 112)	Item-total correlation
Pain^a^				
Item	How bad is your pain today	0.62	-	0.60
Function				
Item 1	Put on a coat	0.83	0.79	0.66
Item 2	Sleep on painful/affected side	0.73	0.75	0.58
Item 3	Wash back/do up bra in back	0.79	0.79	0.60
Item 4	Manage toileting	0.69	0.74	0.52
Item 5	Comb hair	0.82	0.82	0.65
Item 6	Reach high shelf	0.89	0.87	0.73
Item 7	Lift 10 lbs. above shoulder	0.84	0.85	0.62
Item 8	Throw a ball overhand	0.88	0.86	0.70
Item 9	Do usual work	0.83	0.81	0.66
Item 10	Do usual sport	0.53	0.57	0.40
*Diagnostics*				
*χ* ^2^ *; df*		*59.476; 44*	*61.507; 34*	
*RMSEA (90 % CI)*		*0.058 (0.00–0.092)*	*0.085 (0.050–0.118)*	
*p-value RMSEA*		*0.349*	*0.052*	
*TLI; CFI*		*0.942; 0.954*	*0.981; 0.975*	

*df* degrees of freedom, *RMSEA* root mean square error of approximation, *CI* confidence interval, *TLI* Tucker-Lewis index, *CFI* comparative fit index

^a^The Pain item was implemented in a reversed form, with values ranging from 0 = maximum intensity pain to 10 = no pain at all. ^b^In this model, based on a modification index of 11.55 (residual correlation = 0.19) a correlation between items 9 & 10 was allowed (loading = 0.44). Confirmatory factor analysis results and correlations were based on available ASES-p item with full data. Item-total scale correlations were explored with Spearman’s correlation coefficient and were controlled for overlapping

The function and pain subscales presented a substantial correlation r_S_ = 0.605, while 17 % of the patients reported having better function (>25 points) with relatively more pain (≤25 points), or worse function (≤25 p) and less pain (>25 p).

### Validity

In the CFA analysis, factor loadings were >0.50 for all items, with the TLI and CFI coefficients being >0.90 (Table 2). The model including all 11 scale items obtained an RMSEA value of 0.058 (90%CI: 0.00–0.092), while the model considering the 10 function items had an RMSEA = 0.085 (90%CI: 0.050–0.118). In this case, based on a MI = 11.55, a correlation between function items 9 and 10 was also allowed.

The results of the respective Rasch models are presented in Table 3. With the exception of item 6 “reach high shelf” (in both models) and pain (in the first model) all other items had a logit distance >0.10, indicating sufficient spread of item difficulty. Exclusion of the pain item did not change the rank order of the function items, even though in this second model item 6 obtained the same estimation with item 2 “sleep on painful or affected side”. For most items infit and outfit statistics were within the desirable limits. MNSQ values of item 10 were >1.4 and the outfit value for pain was 1.74. The outfit values suggested unexpected subject responses, while the item 10 infit indicated an unexpected response pattern in this item. Nonetheless, no MNSQ value >2.0 was observed in the current data. In both cases, the point-measure correlations were positive, ranging between 0.63 and 0.77, meaning that overall, responses allied with the ability of the subjects. In addition the empirical item-category measures showed that response categories were ordered as expected, and only item 10 had two frequency peaks (at the categories of 0 = unable to do and 2 = somewhat difficult), instead of one. Separation and reliability statistics were acceptable for both models (Table 3). No further factors were suggested by the PCA of the Rasch model residuals, with the 1^st^ contrasts (eigenvalue units) of the 11 and 10-item models being 2.12 and 2.10 respectively.

**Table 3:** Difficulty levels, standard errors, fit statistics and Rank order for two Rasch ASES-p models (*n* = 161)

Item no.	Item description	All ASES-p items^a^					Function items^b^				
		δ (logit)	SE	Infit MNSQ	Outfit MNSQ	Rank order	δ (logit)	SE	Infit MNSQ	Outfit MNSQ	Rank order
Function											
Item 7	Lift 10 lbs above shoulder	1.78	0.13	1.09	0.86	1	1.82	0.14	1.09	0.89	1
Item 8	Throw a ball overhand	1.07	0.13	0.82	0.72	2	1.09	0.13	0.81	0.73	2
Item 3	Wash back/do up bra	0.89	0.12	0.90	0.83	3	0.90	0.13	0.90	0.85	3
Item 2	Sleep on painful side	0.37	0.12	0.98	1.01	4	0.35	0.12	1.02	1.10	4
Item 6	Reach high shelf	0.36	0.12	0.85	0.79	5	0.35	0.12	0.86	0.82	4
Item 10	Do usual sport	−0.12	0.14	1.81	1.78	6	−0.16	0.14	1.86	1.90	5
Item 9	Do usual work	−0.28	0.12	0.73	0.74	7	−0.32	0.13	0.80	0.88	6
Pain											
Item	How bad is your pain today	−0.33	0.12	1.26	1.74	8	-	-	-	-	-
Function											
Item 1	Put on a coat	−0.97	0.13	0.71	0.75	9	−1.05	0.13	0.78	0.89	7
Item 5	Comb hair	−1.28	0.13	0.79	0.78	10	−1.38	0.13	0.81	0.83	8
Item 4	Manage toileting	−1.50	0.13	1.19	1.11	11	−1.61	0.14	1.23	1.16	9
Reliability index: Person/item		0.90/0.98					0.88/0.98				
Separation index: Person/item		2.93/7.53					2.77/8.02				
Variance %: Observed/Expected		59.7/59.5					60.4/60.2				

*δ (logit)* level of item difficulty, based on the Rasch model, *SE* standard error, *MNSQ* mean square fit index, *Rank order* difficulty level, based on the δ (logit) measure. Variance refers to the Rasch model raw variance explained by the measures

^a^In this model, the original pain item was categorized as having 4 response options: 0–1 = No pain; 2–5 = some pain; 6–8 = a lot of pain; 9–10 = maximum pain. ^b^Items 2 and 6 obtained the same δ (logit) estimation and where thus assigned the same rank order

Convergent, divergent and known group validity data are presented in Table 4. The total ASES-p score had correlations r_S_ >0.50 with all CMS versions and with the CMS_O_ components of pain and ADL. As far as the SF-36 scale was concerned, correlations were higher with psychical functioning and role, bodily pain, vitality, and with the PCS component. Lower values were derived for the rest SF-36 dimensions, MCS, and Barthel. A similar tendency was seen in the correlations of the function subscale score, even though the derived coefficients were slightly lower. It is worth highlighting that when this subscale was correlated with the CMS_O_ components, the highest correlation was seen with ADL. Finally, the pain subscale correlated higher with the pain CMS_O_ component, with bodily pain and PCS. Lower correlations were observed with all other measures. As far as the known-group validity was concerned, better health status patients according CMS_O_, PCS, and Barthel also had higher ASES-p scores. The pain subscale did not differentiating well among the three ordered Barthel groups.

**Table 4:** Convergent, divergent and known-group validity of the ASES-p scale with CMS, SF-36 and Barthel

Spearman correlations		ASES-p total (*n* = 106)	Function score (*n* = 112)	Pain score (*n* = 151)
CMS_O_		0.62	0.59	0.45
CMS_R_		0.54	0.49	0.41
CMS_NS_		0.64	0.59	0.47
CMS_O_ component				
Pain		0.62	0.44	0.56
ADL		0.57	0.53	0.45
ROM		0.46	0.45	0.27
Strength		0.37	0.39	0.23
SF-36v2				
Physical functioning		0.59	0.54	0.46
Role Physical		0.60	0.60	0.47
Bodily Pain		0.74	0.67	0.66
General health		0.39	0.37	0.27
Vitality		0.54	0.44	0.40
Social functioning		0.45	0.43	0.35
Role emotional		0.33	0.29	0.34
Mental health		0.39	0.35	0.35
*PCS*		0.65	0.60	0.52
*MCS*		0.32	0.29	0.28
Barthel		0.31	0.33	0.22
Known groups	n			
CMS_O_; median (IQR)				
≤ 30	14–24	23 (15, 38)	10 (5, 22)	15 (10, 25)
31–60	57–83	45 (25, 55)	22 (13, 28)	20 (15, 30)
≥ 61	32–39	63 (48, 79)	34 (27, 39)	30 (20, 45)
*p-value*		<0.0001	<0.0001	<0.0001
PCS; mean (SD)				
< 50	91–134	42 (20)	21 (11)	22 (12)
≥ 50	14–14	76 (16)	36 (10)	39 (12)
*p-value*		<0.0001	<0.0001	<0.0001
Barthel; median (IQR)	n			
80–90	11–15	18 (12, 57)	12 (3, 27)	15 (10,30)
95	12–15	39 (22, 60)	23 (10, 27)	15 (15,30)
100	60–79	50 (42, 68)	27 (18, 33)	25 (15,40)
*p-value*		0.004	0.003	0.019

*ADL* activities of daily living. ASES-p total ranges between 0–100 points. Function and Pain scores range from 0–50 points each. *CMS*_*O*_ Original Constant-Murley score, *CMS*_*R*_ relative CMS standardized for age and sex, *CMS*_*NS*_ CMS score without the strength component: value range 0–75 points, *ADL* activities of daily living, *ROM* range of motion, *PCS and MCS* physical and mental summary components of the SF-36 respectively. Known-group n: corresponds to the min and max subject frequencies per variable category across all three presented scores. *p*-values: three group comparisons were performed with Jonckheere-Terpstra test and two group comparisons with Student’s *t*-test. Analyses were based on available data

### Responsiveness

A total of *n* = 120 patients provided follow-up data. Of those *n* = 10 did not reply the ASES-p pain item and *n* = 50 left unanswered at least one function item. Based on the respective valid replies, scale score differences and standardized effect sizes were higher for improved subjects, compared to non-improved ones. Total and function ASES-p scores, had SES and SRM values around 1, while the pain subscale presented moderate to low effects (Table 5). Correlations between pre-post ASES-p values, for all Table 5 groups, ranged from 0.230 to 0.408, resulting in SRM_Adj_ estimations being almost identical to the SRM ones (results not shown).

**Table 5:** Responsiveness of the ASES-p scale

ASES-p	Number	Baseline mean (SD)	Difference mean (SD)	*p*-value	SES	SRM
Total score (0-100 points)						
All responders	67	46.4 (23.5)	18.9 (25.2)	<0.001	0.80	0.75
Improved	41	50.4 (23.9)	24.4 (23.8)	<0.001	1.02	1.03
Not improved	26	40.0 (21.9)	10.1 (25.3)	0.052	0.46	0.40
Function (0–50 points)						
All responders	70	22.1 (11.8)	10.8 (13.4)	<0.001	0.91	0.80
Improved	42	23.5 (11.8)	13.5 (11.6)	<0.001	1.15	1.17
Not improved	28	20.0 (11.8)	3.8 (14.0)	0.174	0.33	0.27
Pain (0–50 points)						
All responders	110	24.1 (12.9)	6.6 (14.9)	<0.001	0.50	0.45
Improved	55	27.7 (13.0)	9.5 (14.6)	<0.001	0.73	0.65
Not improved	55	20.5 (12.3)	3.7 (14.8)	0.068	0.29	0.25

*SES* standardized effect size, *SRM* standardized response mean, *SD* standard deviation

Differences were derived as follow-up minus baseline values, thus positive differences represent improvement. *P*-values were based on the Student’s paired *t*-test

**Fig. 2:**
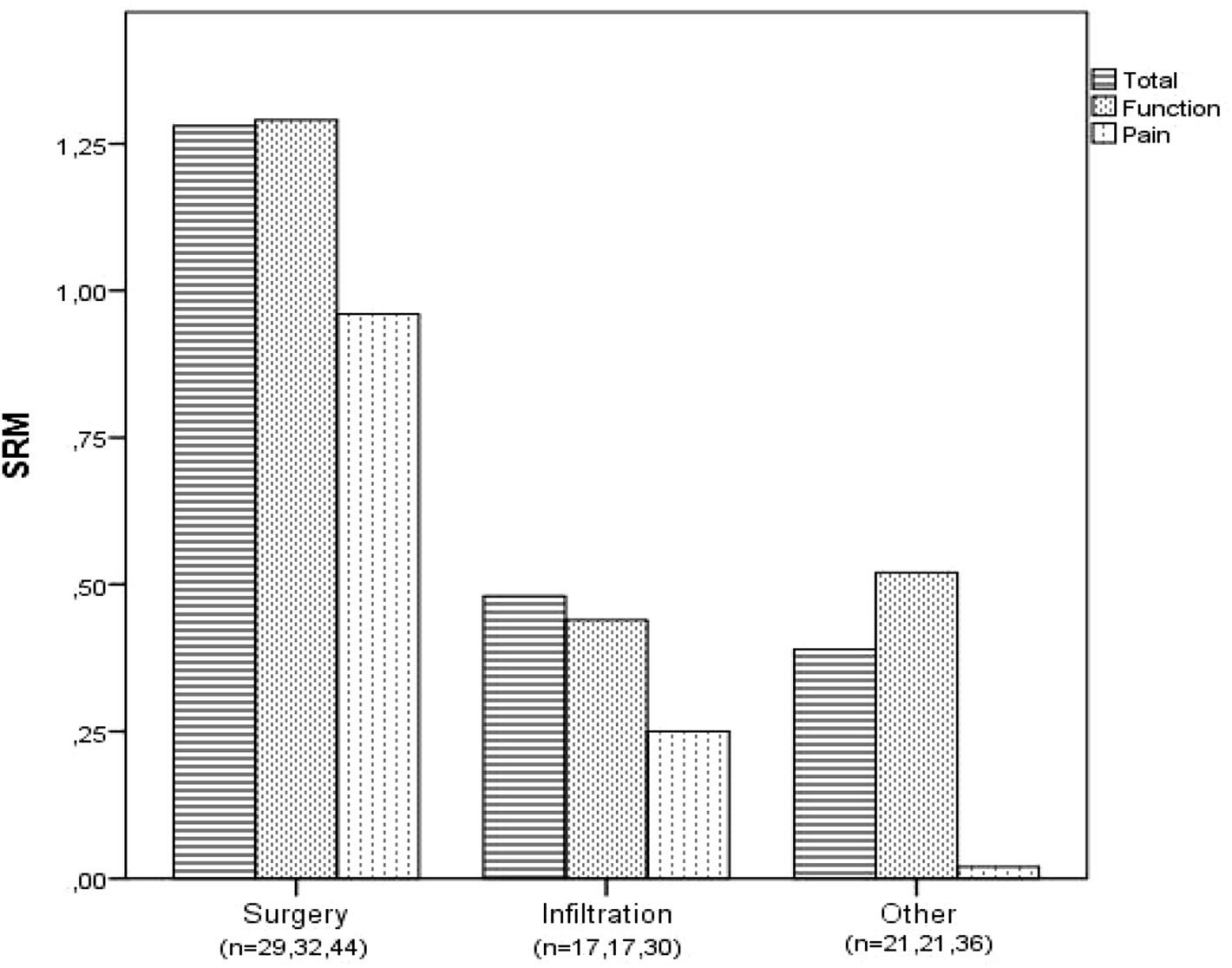
Standardized response means of the ASES-p total, function and pain scores, according received treatment. SRM: standardized response mean. Values below each treatment group (*n* = …) indicate number of valid replies for total scale, function and pain subscale scores respectively

When responsiveness was explored according intervention type, surgery patients has higher standardized effects compared to infiltration and other treatment patients (Fig. 2).

## Discussion

The current study provides data on the validation of the self-administered ASES-p questionnaire into Spanish. The selection of this scale was based on the results of a systematic review and standardized evaluation of HRQoL shoulder instruments, previously performed by our group [19].

Reliability, validity and responsiveness to change were tested considering jointly all 11 scale items and the 10 function items alone, in separate models. Cronbach’s alpha estimations were high and agree with those presented in previous cultural validation studies [11, 13–16, 18], while CFA and Rasch model were applied with overall satisfactory results. CFA factor loadings and most respective fit indexes were acceptable for both tested models, with only the RMSEA value of the 10-function items model being on the borderline. Unidimensionality of the scale was also supported by the Rasch model. None of the 11 items turned out to be unproductive for the measurement scale.

However, the current analyses draw attention on the 10^th^ function item “do usual sports”. This particular item had the highest frequency of baseline missing data; presented lower correlations with two other scale items; had the lowest, even though acceptable, item-total correlation; whereas its distribution did not fit the Rasch model well [34]. Given that the ASES questionnaire was developed in the United States, a question related to doing usual sports may reflect an important ADL in that environment, but not necessarily in ours, at least not for all age groups. According to published information, in our district a high percentage of individuals aged >65 are not involved in regular sport activities [42]. The mean age of item 10 non-responders in the current sample, appear to be in line with this observation. On the other hand, it is worth mentioning that shoulder pathologies do not necessarily inhibit sport activities, given that a wide range of sports are compatible with shoulder problems [43]. This may be an additional reason for the low correlation between the items of pain and doing usual sports, and a possible justification for the misfit of the last item to the Rasch model. Regarding the CFA results, item 10 had the lowest factor loadings in both models, but estimations were still >0.50. Overall item 10 was not degrading, and the current data would not justify its exclusion from the measurement scale [36].

Another interesting finding was the ranking of the pain item, which turned out to be the fourth easier item for the participants. Pain may have been expected to be of higher difficulty, especially considering that it constitutes 50 % the ASES-p total score. However, the baseline pain levels experienced by the study subjects were indeed moderate. It is worth highlighting that this item evaluates current pain intensity (i.e. How bad is your pain today?), meaning that a good or bad day, as far as pain is concerned, could have distorted certain replies. This could explain why some subjects with generally better shoulder status reported higher pain levels than subjects with worse shoulder status, and vice versa. This particular characteristic may have been responsible for the elevated outfit MNSQ of the pain item, which implies detection of outlying responses [34].

In addition, item 7 “lift 10 lbs. above shoulder” and item 8 “throw a ball overhand”, were the most difficult to perform, while item 4 “manage toileting” was the easiest. These findings make clinical sense and offer additional insight on the scale’s construct validity. The first two actions impose an important stress to the shoulder, especially to the mechanisms responsible for its stability. Frequent repetition of these actions increases prevalence of shoulder pathologies [44]. On the other hand, the internal rotations required by the subscapular muscle, for manage toileting, are easier to perform even with the affected side [45]. Previous ASES-p cultural validations, based on response means, presented similar findings for items 7 [14] and 4 [14, 15]. However, factor loadings and discriminatory capacity as evaluated with CFA and the Rasch models are not directly comparable to previous publications. Other authors have implemented principal component and exploratory factor analyses [13], suggesting one [15] and two [13] factor models for the function items alone.

As far as convergent, divergent and known-group validity was concerned, the ASES-p total score presented higher correlations with the different versions of the functional CMS shoulder tool, the pain and ADL components of the latter, as well as with the physical SF-36 dimensions, and PCS. Lower correlations were derived with the ROM and strength CMS_O_ components and the mental SF-36 dimensions. The function subscore behaved in a similar way. The pain subscore correlated higher, as expected, with the pain CMS_O_ component, the PCS and the bodily pain dimension. On the other hand all ASES-p scores presented lower correlations with the Barthel Index. Given that 50 % of the ASES-p total score corresponds to daily life activities, we initially hypothesized that the two instruments would present substantial positive correlations. However, despite having ADL difficulties, most participants were actually BADL independent. This resulted in a small range of Barthel values and consequently low correlations with the scale of interest. On the other hand known-groups validity results were satisfactory and in line with previous hypotheses, including for the Barthel defined groups. Only the ASES-p pain subscore, did not differentiate between the first two Barthel categories. This was not surprising though, given that Barthel does not evaluate pain levels per se [28].

In previous ASES-p cultural validations, convergent and divergent validity were studied with the aid of the Penn [11], DASH or SPADI [13, 14, 18], SST [15] or the OSQ scale [16], while only Yahia et al. [13] did not implement an SF-36 form. The correlations between ASES-p and SF-36, derived from the current data, were similar to the ones presented by Goldhahn et al. [14], but generally higher compared to those presented in the other cultural validations [15].

Responsiveness was also supported by the data. Standardised effect sizes of the total ASES-p score were moderate, but patients with improved self-evaluated ROM and ADL capacity presented higher effect size values compared to the rest. Similar estimations were obtained for the function score. When responsiveness was additionally explored according intervention type, surgery patients had the highest values, while infiltration and other intervention types had low effect sizes. The pain subscore suggested low to moderate responsiveness in all cases.

Certain limitations of this study should be addressed. Our results are based on a sample of public hospitals orthopaedic clinics patients and may not be applicable to shoulder pathology patients in other setting. Also, the current results may not extrapolate to shoulder fracture patients either. Given that most required information was based on self-administered questionnaires, full data was not available for all responders. We refrained from imputing any missing information and only available data were implemented in this study. As far as the CMS instrument was concerned, certain variability across centers existed in the assessment of its strength component. In particular, the weight system used was not always the same. Adjustable dumbbells and weights were used depending on the available resources of the participating centers. For this reason, the four CMS components were also explored separately and a CMS version excluding strength was considered. Finally, no test-retest data is available. This is the subject of another study, currently undertaken by the investigators.

To the best of our knowledge, this is the first extensive validation of the ASES-p scale, implementing both classical and modern test theory. It is also the first in examining the item of pain as part of the scale’s construct. Future studies could focus on the construct validity of ASES-p in different contexts and shoulder pathologies; while the scale’s scoring system is an interesting field for further exploration.

## Conclusions

The presented results, based on classical CFA and Rasch analyses, suggest that the Spanish ASES-p version is a valid and reliable HRQoL tool for shoulder evaluation. Difficulty in doing usual sports was not informative, but neither degrading for the measurement system. Unidimensionality of the scale is supported by the current data.

## Additional file

Additional file 1: Spanish ASES-p version. (PDF 35 kb)

## Abbreviations

ADL: Activities of daily living
ASES-p: Self report section of the American Shoulder and Elbow Surgeons questionnaire
BADL: Basic activities of daily living
CFA: Confirmatory factor analysis
HRQoL: Health related quality of life
CFI: Comparative fit index
CMS: Constant-Murley Score
CMS_NS_: CMS without the strength component
CMS_O_: Original CMS
CMS_R_: Relative CMS
ESSSE: European Society for Surgery of the Shoulder and the Elbow
MCS: Mental component summary of SF-36
MNSQ: Mean square
PCA: Principal component analysis
PCS: Physical component summary of SF-36
RMSEA: Root mean square error of approximation
ROM: Range of movement
SES: Standardized effect size
SF-36: 36-Item Short Form health Survey
SRM: Standardized response mean
SRM_Adj_: Standardized response mean based on pooled standard deviation
TLI: Tucker-Lewis Index
ULSMV: Unweighted list squares estimation method
