# Supplementary material for: Patient self-report section of the ASES questionnaire: a Spanish validation study using classical test theory and the Rasch model
Source: Health Qual Life Outcomes. 2016 Oct 18;14:147. doi: 10.1186/s12955-016-0552-1 (PMC5070228; doi:10.1186/s12955-016-0552-1)
Supplement: Additional file 1: — Spanish ASES-p version. (PDF 35 kb) [file 12955_2016_552_MOESM1_ESM.pdf]

Additional file 1: Spanish ASES-p version

Dolor:

¿Qué intensidad de dolor siente hoy? (haga una marca en la línea)

0 10

Ningún dolor Dolor de intensidad máxima

Función:

Rodee con un circulo el numero que describa su capacidad para realizar las siguientes actividades:

0=Imposible, 1= Con mucha dificultad, 2=Con cierta dificultad, 3= Sin ninguna dificultad

**Actividad**	**Con brazo derecho**	**Con brazo izquierdo**
1. Ponerse un abrigo	0 1 2 3	0 1 2 3
2. Dormir sobre el lado afectado o dolorido	0 1 2 3	0 1 2 3
3. Lavarse la espalda/abrocharse el sujetador por detrás	0 1 2 3	0 1 2 3
4. Limpiarse tras la micción y defecación	0 1 2 3	0 1 2 3
5. Peinarse	0 1 2 3	0 1 2 3
6. Llegar a una balda alta	0 1 2 3	0 1 2 3
7. Levantar 4,5 kg. por encima del hombro (ej. 3 botellas de agua de 1,5 kg.)	0 1 2 3	0 1 2 3
8. lanzar una pelota por encima de la cabeza (ej. Saque de banda, tirar una piedra)	0 1 2 3	0 1 2 3
9. Realizar su trabajo habitual	0 1 2 3	0 1 2 3
10. Realizar su deporte habitual	0 1 2 3	0 1 2 3
